# A Multiplex SYBR Green Real-Time PCR Assay for the Detection of Three Colistin Resistance Genes from Cultured Bacteria, Feces, and Environment Samples

**DOI:** 10.3389/fmicb.2017.02078

**Published:** 2017-10-27

**Authors:** Jiyun Li, Xiaomin Shi, Wenjuan Yin, Yang Wang, Zhangqi Shen, Shuangyang Ding, Shaolin Wang

**Affiliations:** ^1^Beijing Advanced Innovation Center for Food Nutrition and Human Health, College of Veterinary Medicine, China Agricultural University, Beijing, China; ^2^Beijing Key Laboratory of Detection Technology for Animal-Derived Food Safety and Beijing Laboratory for Food Quality and Safety, Beijing, China

**Keywords:** colistin resistance, *mcr-1*, *mcr-2*, *mcr-3*, real-time PCR

## Abstract

The aim of the study was to develop a multiplex assay for rapid detection of *mcr-1, mcr-2*, and *mcr-3*, a group of genes of conferring resistance to colistin mediated by plasmid in Enterobacteriaceae. A SYBR Green based real-time PCR assay has been designed to detect the *mcr* genes, and applied to cultured bacteria, feces and soil samples. All three *mcr* genes could be detected with a lower limit of 10^2^ cultured bacteria. This test was highly specific and sensitive, and generated no false-positive results. The assay was also conclusive when applied to feces and soil samples containing *mcr-1*-positive *Escherichia coli*, which could facilitate the screening of *mcr* genes not only in the bacteria, but also directly from the environment. This simple, rapid, sensitive, and specific multiplex assay will be useful for rapid screening of the colistin resistance in both clinical medicine and animal husbandry.

## Introduction

Colistin is regarded as the last antibiotic for the treatment of infection of Carbapenem Resistant Enterobacteriaceae (CRE). Since the discovery of first plasmid-mediated colistin resistance gene *mcr-1* in 2015 (Liu et al., 2016), it has caught worldwide attention. The *mcr-1* gene has been widely reported from all major continents, North America, North Africa, Southeast Asia, and Europe (Wang et al., 2017a). Shortly after that, six variants (*mcr-1.2* to *mcr-1.7*; Figure S1) of *mcr-1* with one amino acid change have been reported and submitted to GeneBank (https://www.ncbi.nlm.nih.gov/gene). In 2016, a novel colistin resistance gene, *mcr-2*, was discovered from Belgium with 76.75% identity to *mcr-1*. Very recently, another novel colistin resistance gene, *mcr-3*, has been discovered with much higher variation comparing to *mcr-1* and *mcr-2* (Xavier et al., 2016; Yin et al., 2017). All three *mcr* genes could be disseminated through the plasmid, which further facilitates the dissemination of colistin resistance not only in the clinical setting (Wang et al., 2017a), but also widely in the animal husbandry and various environment (Hembach et al., 2017; Huang et al., 2017; Wang et al., 2017b; Zhou et al., 2017). This poses a serious threat to the public health and animal husbandry, and it is urgent to develop a method to detect three *mcr* genes simultaneously not only in the bacterial culture from clinical samples, but also environment samples. However, the conventional detection method, such as conventional PCR and Sanger sequencing, is time-assuming and labor intensive. It's necessary to develop a rapid multiplex real-time PCR assay to detect the three colistin resistance gene simultaneously. Recently several real-time PCR method for *mcr-1* detection has been reported (Bontron et al., 2016; Dona et al., 2017) but none for *mcr-2* and *mcr-3*. Here, we develop a fast, sensitive Real-time PCR method for specific detection of three plasmid-mediated colistin resistances.

## Validation of the method

### Real-time PCR primer design and synthesis of *mcr-2*

Primer premier 5.0 software (Biosoft International, Palo Alto, CA) was used to design the specific real-time PCR primers for three colistin resistance genes (*mcr-1, mcr-2, mcr-3*; Table 1). The sequences of primers were searched against the NCBI database to confirm the specificity of the primers using Primer-BLAST module (https://www.ncbi.nlm.nih.gov/tools/primer-blast/). Conventional PCR was used to evaluate the specificity of the primers. Due to the lacking of *mcr-2* carrying isolate in our laboratory, the complete *mcr-2* gene was synthetized according to the sequence (GenBank accession no.LT598652).

**Table 1:** Primers for detection of the *mcr-1, mcr-2*, and *mcr-3* gene.

	**Primer**	**Sequence(5′ → 3′)**	**Gene**	**Product length(bp)**	**References**
Regular PCR	CLR5-F	CGGTCAGTCCGTTTGTTC	MCR-1	309	Liu et al., 2016
	CLR5-R	CTTGGTCGGTCTGTAGGG			
	MCR2-IF	TGTTGCTTGTGCCGATTGGA	MCR-2	567	Xavier et al., 2016
	MCR2-IR	AGATGGTATTGTTGGTTGCTG			
	MCR3-F	TTGGCACTGTATTTTGCATTT	MCR-3	542	Yin et al., 2017
	MCR3-R	TTAACGAAATTGGCTGGAACA			
Real-time PCR	mcr1-qf	AAAGACGCGGTACAAGCAAC	MCR-1	213	This study
	mcr1-qr	GCTGAACATACACGGCACAG			
	mcr2-qf	CGACCAAGCCGAGTCTAAGG	MCR-2	92	This study
	mcr2-qr	CAACTGCGACCAACACACTT			
	mcr3-qf	ACCTCCAGCGTGAGATTGTTCCA	MCR-3	169	This study
	mcr3-qr	GCGGTTTCACCAACGACCAGAA			

### The standard curve for *mcr-1/2/3*

All three *mcr* genes were cloned into the PMD19-T vector, and then transferred into the DH5a cell. The constructed plasmids were extracted using the PureYield™ Plasmid Midiprep System (Promega) according to the manufactures instruction. A serial dilution of plasmid DNA concentration was conducted to establish the standard curve using the real-time PCR assay on the QuantStudio™ 7 Flex Real-Time PCR System (Applied Biosystems). The reaction was prepared by mixing the SYBR™ Green master mix (Applied Biosystems) with primers and template plasmid DNA. The real-time PCR condition was programmed as follows: a cycle of 50°C for 2min, 95°C for 3min, then 40 cycles of 95°C for 30s, 60°C for 30s, and 72°C for 30s, followed by a ramp from 72 to 95°C for melting curve stage.

### Establishment of quantitative PCR method

**Figure 1:**
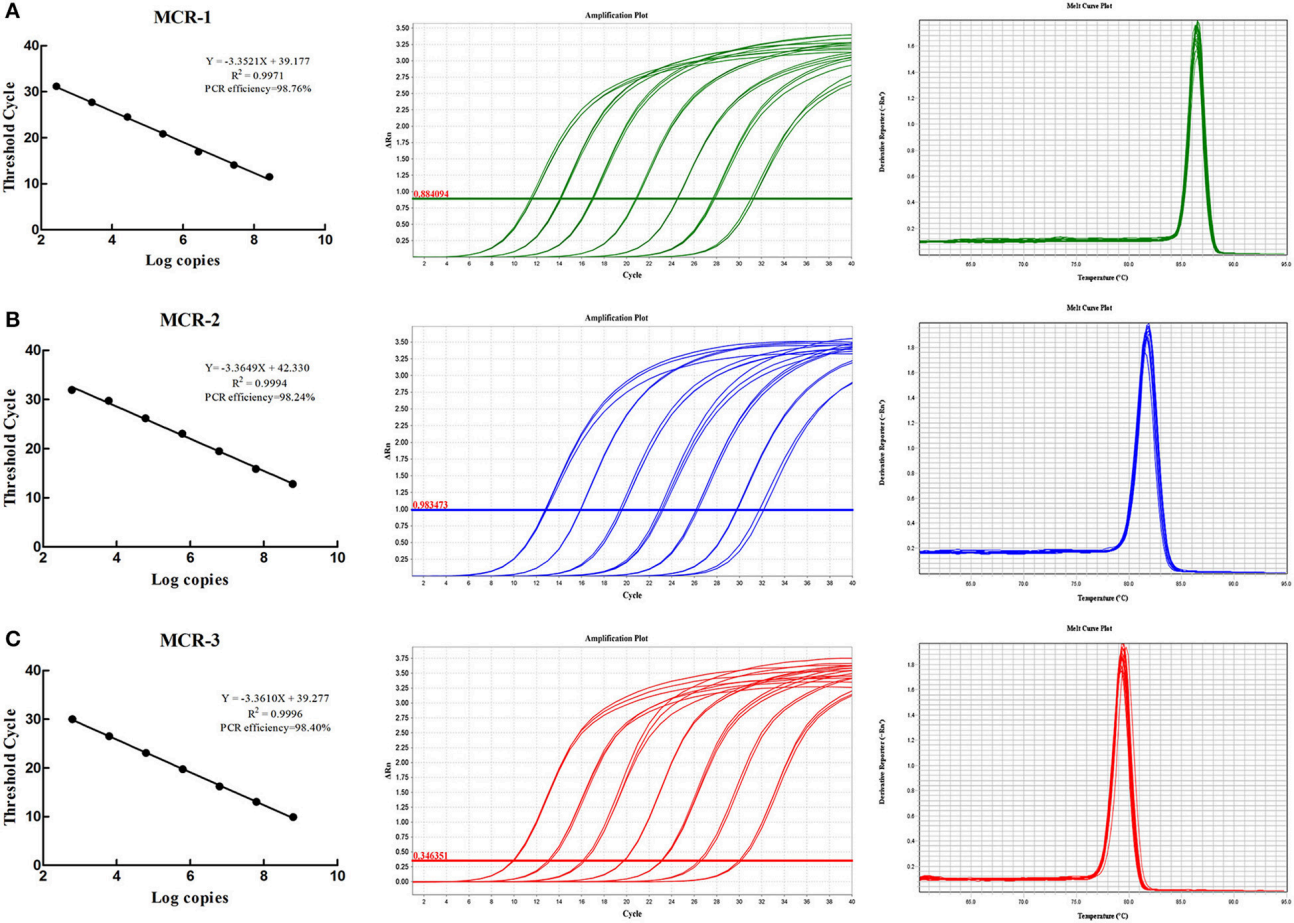
Real-time PCR standard curves, amplification curves and melting curves**. (A–C)** Show real-time PCR standard curves, amplification curves and melting curves for *mcr-1, mcr-2*, and *mcr-3*.

Once the primer design finished, all primers were evaluated *in silico* to confirm the specificity, and there is no other sequences match these primers except *mcr-1, mcr-2*, and *mcr-3* genes, respectively. Moreover, the conventional PCR was used to confirm the specificity of primers, and the results indicated the high specificity of primers (Figure S2). The assay linearity and limit of detection were conducted using a serial dilution of the recombinant plasmids pMCR-1, pMCR-2, pMCR-3 carrying the *mcr-1, mcr-2, mcr-3* gene. The detection of copies range was 2.7 × 10^2^~2.7 × 10^8^, 6.1 × 10^2^~6.1 × 10^8^, 6.3 × 10^2^~6.3 × 10^8^ for *mcr-1, mcr-2, mcr-3* gene, and Ct (Cycle threshold) range were 31.18~11.50, 32.63~12.82, 30.02~9.90 for *mcr-1, mcr-2, mcr-3* genes. All *r*^2^ values were >0.997 and all amplification efficiency were >98% (Figure 1). The formula for absolute quantification was shown in Figure 1.

## Application and effectiveness of the method

To validate the method, some *E. coli* isolates from animal and human origin (Table 2), were selected to conduct the real-time PCR assay for screening. The PowerSoil® DNA Isolation kit (MOBIO, USA) was used to the metagenomic DNA from feces and soil for the validation of methods. The absolute copy number of *mcr-1* in the feces and soil samples were quantified by using the standard curve and Ct value.

**Table 2:** Detection of the *mcr-1, mcr-2*, and *mcr-3* gene in isolates.

**Isolate**	**Origin**	**Species**	**Genetype**	**Gene location**	**Real-time PCR for MCR**			
					***mcr-1***	***mcr-2***	***mcr-3***	**CT ±*SD***
ATCC25922	–	*E. coli*	None	–	–	–	–	Undetermined
DH5a-*mcr-1*	–	*E. coli*	*mcr-1*	Plasmid	+	–	–	12.8 ± 0.18
DH5a-*mcr-2*	–	*E. coli*	*mcr-2*	Plasmid	–	+	–	9.62 ± 0.09
DH5a-*mcr-3*	–	*E. coli*	*mcr-3*	Plasmid	–	–	+	18.7 ± 0.01
E1	Chicken	*E. coli*	*mcr-1*	Chromosome	+	–	–	12.8 ± 0.18
E2	Chicken	*E. coli*	*mcr-1*	Chromosome	+	–	–	14.5 ± 0.19
E3	Pig	*E. coli*	*mcr-1*	Chromosome	+	–	–	14.1 ± 0.05
E4	Pig	*E. coli*	*mcr-1*	Chromosome	+	–	–	13.8 ± 0.35
E5	Pig	*E. coli*	*mcr-3*	Plasmid	–	–	+	16.7 ± 0.22
E6	Pig	*E. coli*	*mcr-1*	Plasmid	+	–	–	13.8 ± 0.08
E7	Pig	*E. coli*	*mcr-3*	Plasmid	–	–	+	15.6 ± 0.03
E8	Pig	*E. coli*	*mcr-3*	Plasmid	–	–	+	20.4 ± 0.04
E9	Pig	*E. coli*	*mcr-3*	Plasmid	–	–	+	13.3 ± 0.03
E10	Pig	*E. coli*	*mcr-3*	Plasmid	–	–	+	16.7 ± 0.02
E11	Pig	*E. coli*	*mcr-3*	Plasmid	–	–	+	14.6 ± 0.02
E12	Pig	*E. coli*	*mcr-3*	Plasmid	–	–	+	16.1 ± 0.15
E13	Pig	*E. coli*	*mcr-3*	Plasmid	–	–	+	17.6 ± 0.26
E14	Pig	*E. coli*	*mcr-1*	Plasmid	+	–	–	14.8 ± 0.02
E15	Pig	*E. coli*	*mcr-1*	Plasmid	+	–	–	15.4 ± 0.06
E16	Pig	*E. coli*	*mcr-1*	Plasmid	+	–	–	14.7 ± 0.23
E17	Pig	*E. coli*	*mcr-1*	Plasmid	+	–	–	13.3 ± 0.29
E18	Pig	*E. coli*	*mcr-1*	Plasmid	+	–	–	12.7 ± 0.05
E19	Pig	*E. coli*	*mcr-1*	Plasmid	+	–	–	15.9 ± 0.05
E20	Pig	*E. coli*	*mcr-1*	Plasmid	+	–	–	15.3 ± 0.29
E21	Pig	*E. coli*	*mcr-1*	Plasmid	+	–	–	12.6 ± 0.15
E22	Pig	*E. coli*	*mcr-1*	Plasmid	+	–	–	16.7 ± 0.07
E23	Pig	*E. coli*	*mcr-1*	Plasmid	+	–	–	15.2 ± 0.07
E24	Pig	*E. coli*	*mcr-1*	Plasmid	+	–	–	12.6 ± 0.09
KP1	Human	*K. pneumoniae*	*mcr-1*	Plasmid	+	–	–	15.4 ± 0.06

**Figure 2:**
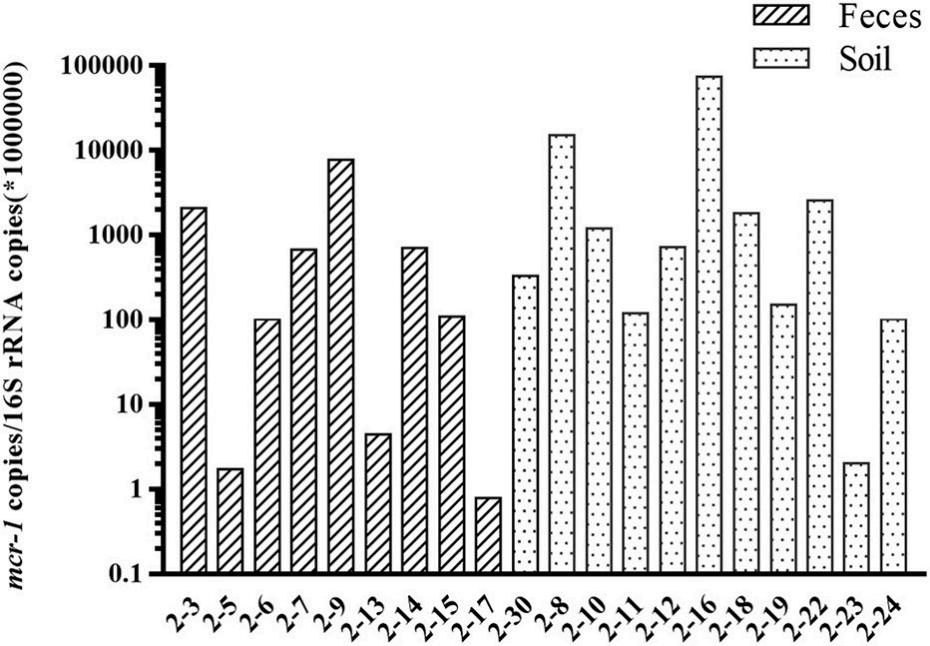
Detection of *mcr-1* gene in soil and feces samples. The relative abundance of *mcr-1* gene (*mcr-1* copies /per 1,000,000 copies of 16S rRNA) in the soil and feces samples.

A total of 25 field isolates were selected to evaluate the specificity of the primer. Seventeen *mcr-1* positive strains, and eight *mcr-3* positive strains of different origins were tested using multiplex real-time PCR assay. *E. coli* ATCC25922 was used as negative control strain, and three DH-5α strains carrying *mcr* genes were used as positive control. Three independent technical replicates were applied to each sample. The results of real-time PCR assay showed 100% concordance with previous conventional PCR results. The Ct range were 12.6~16.7, 9.62, 13.3~20.4 for *mcr-1, mcr-2, mcr-3* genes (Table 2). The method was also used to further validation to screen the *mcr-1* in the feces and soil samples. A total of 20 feces and soil samples (three independent technical replicates) from chicken farm were selected for the evaluation of the method, the real-time PCR assay showed that the copies of *mcr-1* gene ranges from 1~10^5^ (normalized using 16s rRNA from 0.25 g dry feces or soil samples) in the metagenomic DNA from feces and soil samples (Figure 2).

## Advantages and limitations

Since the first discovery of *mcr-1*, multiple variants have been identified with either single nucleotide variation (*mcr-1.2*~*1.7*) or relatively less conserved, especially *mcr-3*. The prevalence of *mcr* genes is rising very fast in the clinical medicine (Wang et al., 2017a). Thus, the rapid screening methods are required for the detection of these *mcr* genes. So far, there are two reports about fast screening of *mcr-1* using the real-time PCR method, which has been validated using both pure bacteria and the human stool samples (Bontron et al., 2016; Dona et al., 2017). Both reports have approved the effectiveness of the real-time method, but only can be used for the screening of *mcr-1*. With the growing number of novel *mcr* gene variants discovered, especially *mcr-2* and *mcr-3*, a multiplex assay is necessary for screening all three types of *mcr* genes from various samples. The method developed in this study is very effective for the screening of all three *mcr* genes, not only in the cultured bacteria, but also directly from feces and soil samples. The *mcr-1* can be detected as low as 1 copy per 1,000,000 copies of 16s rRNA in the feces samples, and the highest abundance of *mcr-1* could reach 10^5^ copies per 1,000,000 copies of 16s rRNA (Figure 2). Relative abundance was the most popular method to measure the abundance of antibiotic resistance genes in the feces and soil samples, as the composition and abundance of microbiome varies a lot among different feces and soil samples (Bontron et al., 2016; Subirats et al., 2017). In this study, both *mcr-1* and *mcr-3* has been successfully detected in the cultured bacteria from natural animal and environment isolates. However, *mcr-2* has not been validated in the cultured bacteria, as lacking *mcr-2* carrying bacteria in our lab. The specificity of the primers has been confirmed by both traditional PCR and melting curve analysis. The limitation of this method was all three *mcr* genes could not be detected in one reaction comparing with the Taqman assay. However, this method does have the flexibility for screening different *mcr* genes with any combination at relative lower cost, as the co-existence of *mcr* genes has not been reported in the clinical medicine, and it is not necessary to screen all three mcr genes. Recently, only one case was reported about the co-occurrence of *mcr-1* and *mcr-3* in one *E. coli* isolate from cattle farm (Hernández et al., 2017). Also considering the number of novel *mcr* gene has been growing, the flexible combination of the detection assay does have its advantage.

In conclusion, this SYBR Green-based real-time PCR assay is a rapid, sensitive, and highly specific detection assay for the *mcr-1, mcr-2, and mcr-3* genes either from cultured bacteria, feces, and soil samples. It is easy to perform in any laboratory having at its disposal a qPCR machine. This rapid technique may be used for the evaluation of the prevalence of this resistance trait in humans and animals (surveillance studies). In addition, it will be a valuable tool for fast screening and quantifying all *mcr* genes not only in the bacterial, but also in feces and soil samples.

## Author contributions

Conceived and designed the experiments: SW, YW, ZS. Performed the experiments: JL, XS, WY. Analyzed the data: SW, JL, XS. Wrote the paper: SW, JL, WY, SD.

### Conflict of interest statement

The authors declare that the research was conducted in the absence of any commercial or financial relationships that could be construed as a potential conflict of interest.

## Supplementary material

Figure S1 Multiple sequence alignment of *mcr-1* variants.

Figure S2 Conventional PCR amplification and real-time PCR melting curve for *mcr* genes. **(A–C)** were the electrophoresis gel (left) and melting curve (right) of *mcr-1, mcr-2*, and *mcr-3*. M: Marker. NTC: negative control. 1A, 1B, 1C, 1D were *mcr-1* positive strains. 2A was *mcr-2* positive strain. 3A, 3B, 3C, 3D were *mcr-3* positive strains. 1A, 2A, 3A were identical templates in this figure. 2B, 2C, 2D were negative strains.

## References

[B1] Bontron S. Poirel L. Nordmann P. (2016). Real-time PCR for detection of plasmid-mediated polymyxin resistance (mcr-1) from cultured bacteria and stools. J. Antimicrob. Chemother. 71, 2318–2320. 10.1093/jac/dkw139 27121402

[B2] Donà V. Bernasconi O. J. Kasraian S. Tinguely R. Endimiani A. (2017). A SYBR(R) Green-based real-time PCR method for improved detection of mcr-1-mediated colistin resistance in human stool samples. J. Glob. Antimicrob. Resist. 9, 57–60. 10.1016/j.jgar.2017.01.007 28400211

[B3] Hembach N. Schmid F. Alexander J. Hiller C. Rogall E. T. Schwartz T. (2017). Occurrence of the mcr-1 colistin resistance gene and other clinically relevant antibiotic resistance genes in microbial populations at different municipal wastewater treatment plants in germany. Front. Microbiol. 8:1282. 10.3389/fmicb.2017.01282 28744270 PMC5504345

[B4] Hernández M. Iglesias M. R. Rodríguez-Lázaro D. Gallardo A. Quijada N. Miguela-Villoldo P.. (2017). Co-occurrence of colistin-resistance genes mcr-1 and mcr-3 among multidrug-resistant *Escherichia coli* isolated from cattle, Spain, September 2015. Euro Surveill. 22:30586. 10.2807/1560-7917.ES.2017.22.31.30586 28797328 PMC5553059

[B5] Huang X. Yu L. Chen X. Zhi C. Yao X. Liu Y.. (2017). High prevalence of colistin resistance and mcr-1 gene in *Escherichia coli* isolated from food animals in china. Front. Microbiol. 8:562. 10.3389/fmicb.2017.00562 28421056 PMC5378783

[B6] Liu Y. Y. Wang Y. Walsh T. R. Yi L. X. Zhang R. Spencer J.. (2016). Emergence of plasmid-mediated colistin resistance mechanism MCR-1 in animals and human beings in China: a microbiological and molecular biological study. Lancet Infect. Dis. 16, 161–168. 10.1016/S1473-3099(15)00424-7 26603172

[B7] Subirats J. Royo E. Balcázar J. L. Borrego C. M. (2017). Real-time PCR assays for the detection and quantification of carbapenemase genes (bla KPC, bla NDM, and bla OXA-48) in environmental samples. Environ. Sci. Pollut. Res. Int. 24, 6710–6714. 10.1007/s11356-017-8426-6 28084599

[B8] Wang Y. Tian G. B. Zhang R. Shen Y. Tyrrell J. M. Huang X.. (2017a). Prevalence, risk factors, outcomes, and molecular epidemiology of mcr-1-positive Enterobacteriaceae in patients and healthy adults from China: an epidemiological and clinical study. Lancet Infect. Dis. 17, 390–399. 10.1016/S1473-3099(16)30527-8 28139431

[B9] Wang Y. Zhang R. Li J. Wu Z. Yin W. Schwarz S.. (2017b). Comprehensive resistome analysis reveals the prevalence of NDM and MCR-1 in Chinese poultry production. Nat. Microbiol. 2:16260. 10.1038/nmicrobiol.2016.260 28165472

[B10] Xavier B. B. Lammens C. Ruhal R. Kumar-Singh S. Butaye P. Goossens H.. (2016). Identification of a novel plasmid-mediated colistin-resistance gene, *mcr-2*, in *Escherichia coli*, Belgium, June 2016. Euro Surveill. 21:30280. 10.2807/1560-7917.ES.2016.21.27.30280 27416987

[B11] Yin W. Li H. Shen Y. Liu Z. Wang S. Shen Z.. (2017). Novel plasmid-mediated colistin resistance gene *mcr-3* in *Escherichia coli*. MBio 8:e00543-17. 10.1128/mBio.00543-17 28655818 PMC5487729

[B12] Zhou H.-W. Zhang T. Ma J.-H. Fang Y. Wang H.-Y. Huang Z.-X.. (2017). Occurrence of plasmid- and chromosome-carried *mcr-1* in waterborne Enterobacteriaceae in China. Antimicrob. Agents Chemother. 61:e00017-17. 10.1128/AAC.00017-17 28559252 PMC5527621

