# Supplementary material for: A Multiplex SYBR Green Real-Time PCR Assay for the Detection of Three Colistin Resistance Genes from Cultured Bacteria, Feces, and Environment Samples
Source: Front Microbiol. 2017 Oct 27;8:2078. doi: 10.3389/fmicb.2017.02078 (PMC5663727; doi:10.3389/fmicb.2017.02078)
Supplement: Figure S1 — Multiple sequence alignment of mcr-1 variants. [file Image1.PDF]

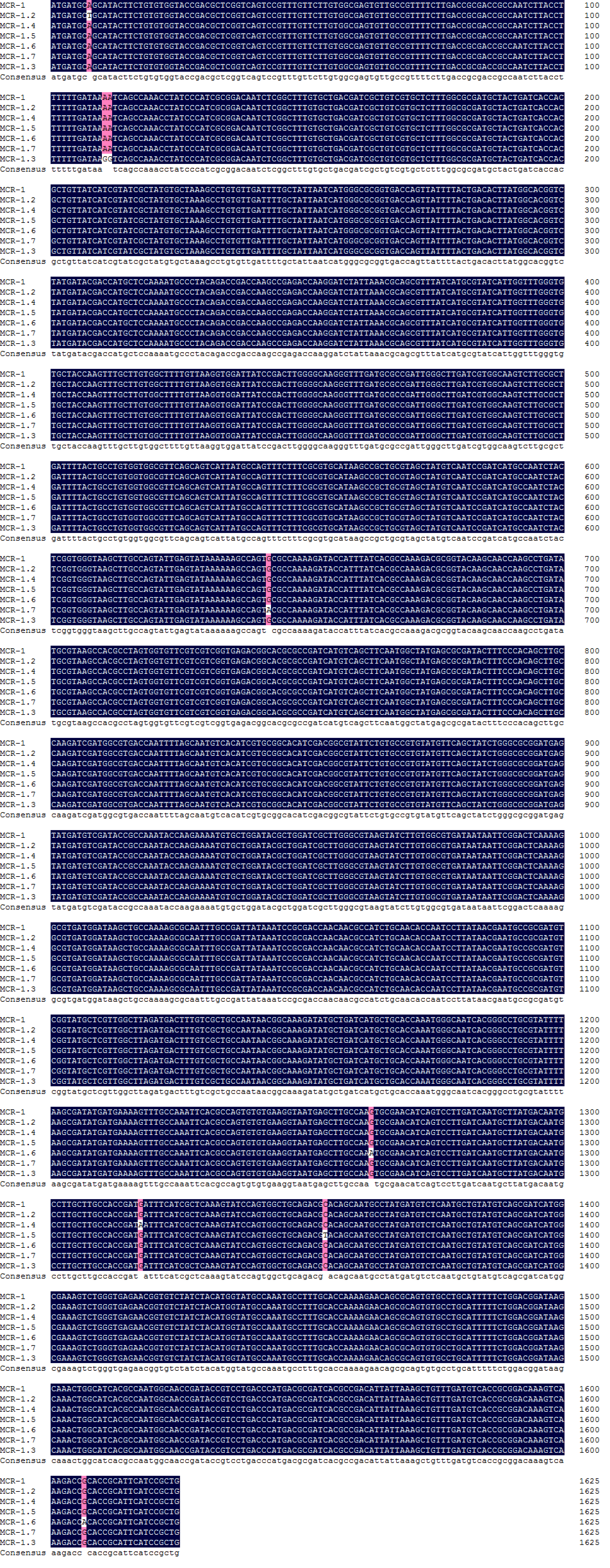
MCR-1
MCR-1.2
MCR-1.4
MCR-1.5
MCR-1.6
MCR-1.7
MCR-1.3
Consensus atgatgc gcatacttctgtgtggtaccgacgctcggtcagtccgtttgttcttgtggcgagtgttgccgttttcttgaccgcgaccgccaatcttacct 100
Consensus ttttgataa tcagccaaacctatcccatcgcggacaatctcggctttgtgctgacgatcgctgtcgtgctctttggcgcgatgctactgatcaccac 200
Consensus gctgttatcatcgtatcgctatgtgctaaagcctgtgttgattttgctattaatcatgggcgcggtgaccagttattttactgacacttatggcacggtc 300
Consensus tatgatacgaccatgctccaaaatgccctacagaccgaccaagccgagaccaaggatctattaaacgcagcgtttatcatgcgtatcattggtttgggtg 400
Consensus tgctaccaagtttgcttgtggcttttgttaaggtggattatccgacttggggcaagggtttgatgcgccgattgggcttgatcgtggcaagtcttgcgct 500
Consensus gattttactgcctgtggtggcgttcagcagtcattatgccagtttctttcgcgtgcataagccgctgcgtagctatgtcaatccgatcatgccaatctac 600
Consensus tcggtgggtaagcttgccagtattgagtataaaaaagccagt cgccaaaagataccatttatcacgccaaagacgcggtacaagcaacaagcctgata 700
Consensus tgcgtaagccacgcctagtggtgttcgtcgtcggtgagacggcacgcgccgatcatgtcagcttcaatggctatgagcgcgatactttcccacagcttgc 800
Consensus caagatcgatggcgtgaccaattttagcaatgtcacatcgtgcggcacatcgacggcgtattctgtgccgtgtatgttcagctatctgggcgcggatgag 900
Consensus tatgatgtcgataccgccaaataccaagaaaatgtgctggatacgctggatcgcttgggcgtaagtatcttgtggcgtgataataattcggactcaaaag 1000
Consensus gcgtgatggataagctgccaaaagcgcaatttgccgattataaatccgcgaccaacaacgccatctgcaacaccaatccttataacgaatgccgcgatgt 1100
Consensus cggtatgctcgttggcttagatgactttgtcgctgccaataacggcaaagatatgctgatcatgctgcaccaaatgggcaatcacgggcctgcgtatttt 1200
Consensus aagcgatatgatgaaaagtttgccaaattcacgccagtgtgtgaaggtaatgagcttgccaa tgcgaacatcagtccttgatcaatgcttatgacaatg 1300
Consensus ccttgcttgccaccgat atttcatcgctcaaagtatccagtggctgcagacg acagcaatgcctatgatgtctcaatgctgtatgtcagcgatcatgg 1400
Consensus cgaaagtctgggtgagaacggtgtctatctacatggtatgccaaatgcctttgcaccaaaagaacagcgcagtgtgcctgcatttttctggacggataag 1500
Consensus caaactggcatcacgccaatggcaaccgataccgtcctgacccatgacgcgatcacgccgacattattaaagctgtttgatgtcaccgcggacaaagtca 1600
Consensus aagacc caccgcattcatccgctg 1625
