# Supplementary figures and images for: A Multiplex SYBR Green Real-Time PCR Assay for the Detection of Three Colistin Resistance Genes from Cultured Bacteria, Feces, and Environment Samples
Source: Front Microbiol. 2017 Oct 27;8:2078. doi: 10.3389/fmicb.2017.02078 (PMC5663727; doi:10.3389/fmicb.2017.02078)

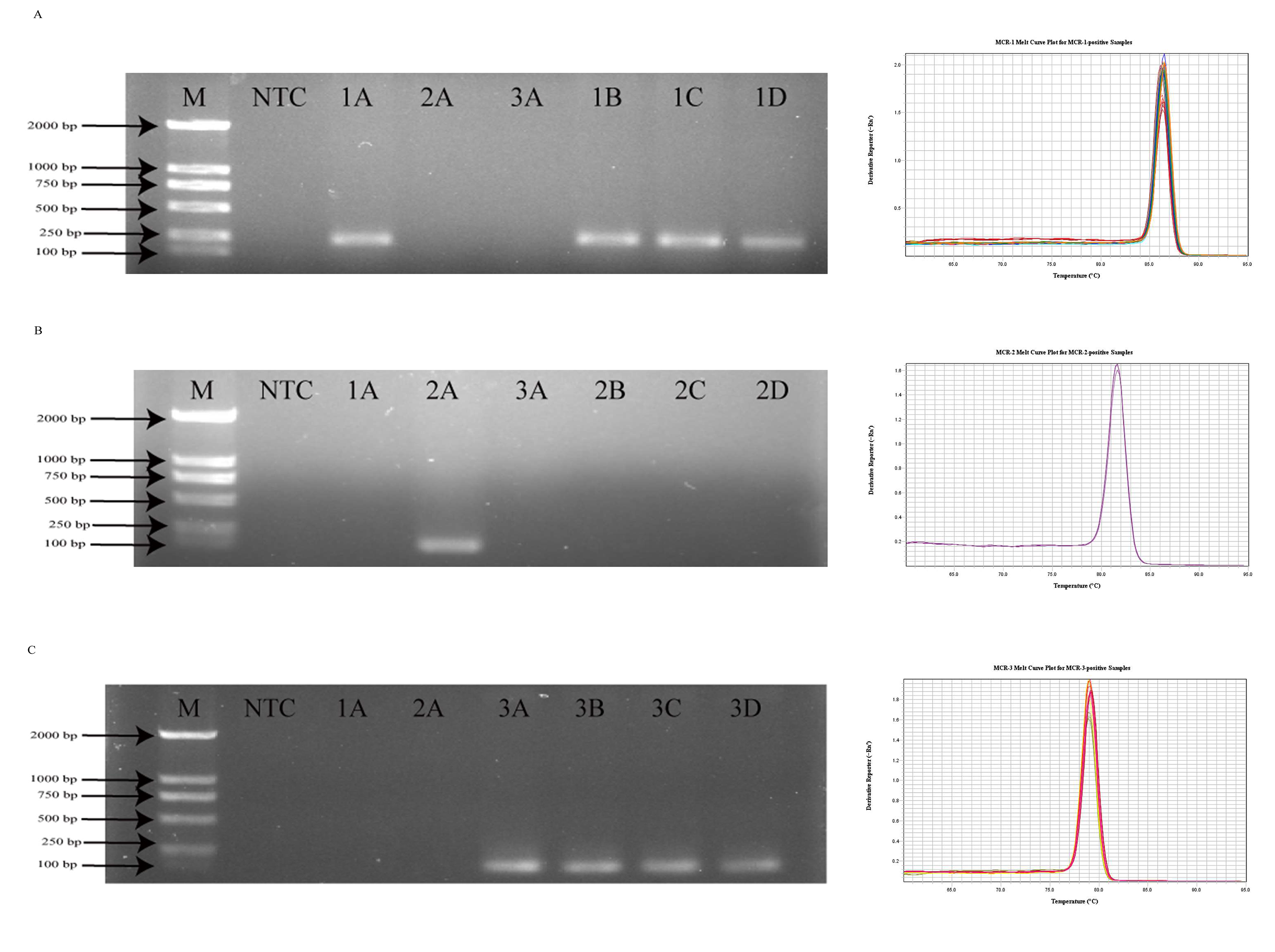

Supplement: Figure S2 — Conventional PCR amplification and real-time PCR melting curve for mcr genes. (A–C) were the electrophoresis gel (left) and melting curve (right) of mcr-1, mcr-2, and mcr-3. M: Marker. NTC: negative control. 1A, 1B, 1C, 1D were mcr-1 positive strains. 2A was mcr-2 positive strain. 3A, 3B, 3C, 3D were mcr-3 positive strains. 1A, 2A, 3A were identical templates in this figure. 2B, 2C, 2D were negative strains. [file Image2.TIF]
